# Macrophage Polarization and Its Role in Liver Disease

**DOI:** 10.3389/fimmu.2021.803037

**Published:** 2021-12-14

**Authors:** Cheng Wang, Cheng Ma, Lihong Gong, Yuqin Guo, Ke Fu, Yafang Zhang, Honglin Zhou, Yunxia Li

**Affiliations:** State Key Laboratory of Southwestern Chinese Medicine Resources, Key Laboratory of Standardization for Chinese Herbal Medicine, Ministry of Education, School of Pharmacy, Chengdu University of Traditional Chinese Medicine, Chengdu, China

**Keywords:** macrophage polarization, liver disease, acute liver injury, viral hepatitis, alcoholic liver disease, metabolic-associated fatty liver disease, liver fibrosis, hepatocellular carcinoma

## Abstract

Macrophages are important immune cells in innate immunity, and have remarkable heterogeneity and polarization. Under pathological conditions, in addition to the resident macrophages, other macrophages are also recruited to the diseased tissues, and polarize to various phenotypes (mainly M1 and M2) under the stimulation of various factors in the microenvironment, thus playing different roles and functions. Liver diseases are hepatic pathological changes caused by a variety of pathogenic factors (viruses, alcohol, drugs, etc.), including acute liver injury, viral hepatitis, alcoholic liver disease, metabolic-associated fatty liver disease, liver fibrosis, and hepatocellular carcinoma. Recent studies have shown that macrophage polarization plays an important role in the initiation and development of liver diseases. However, because both macrophage polarization and the pathogenesis of liver diseases are complex, the role and mechanism of macrophage polarization in liver diseases need to be further clarified. Therefore, the origin of hepatic macrophages, and the phenotypes and mechanisms of macrophage polarization are reviewed first in this paper. It is found that macrophage polarization involves several molecular mechanisms, mainly including TLR4/NF-κB, JAK/STATs, TGF-β/Smads, PPARγ, Notch, and miRNA signaling pathways. In addition, this paper also expounds the role and mechanism of macrophage polarization in various liver diseases, which aims to provide references for further research of macrophage polarization in liver diseases, contributing to the therapeutic strategy of ameliorating liver diseases by modulating macrophage polarization.

**Graphical Abstract:**
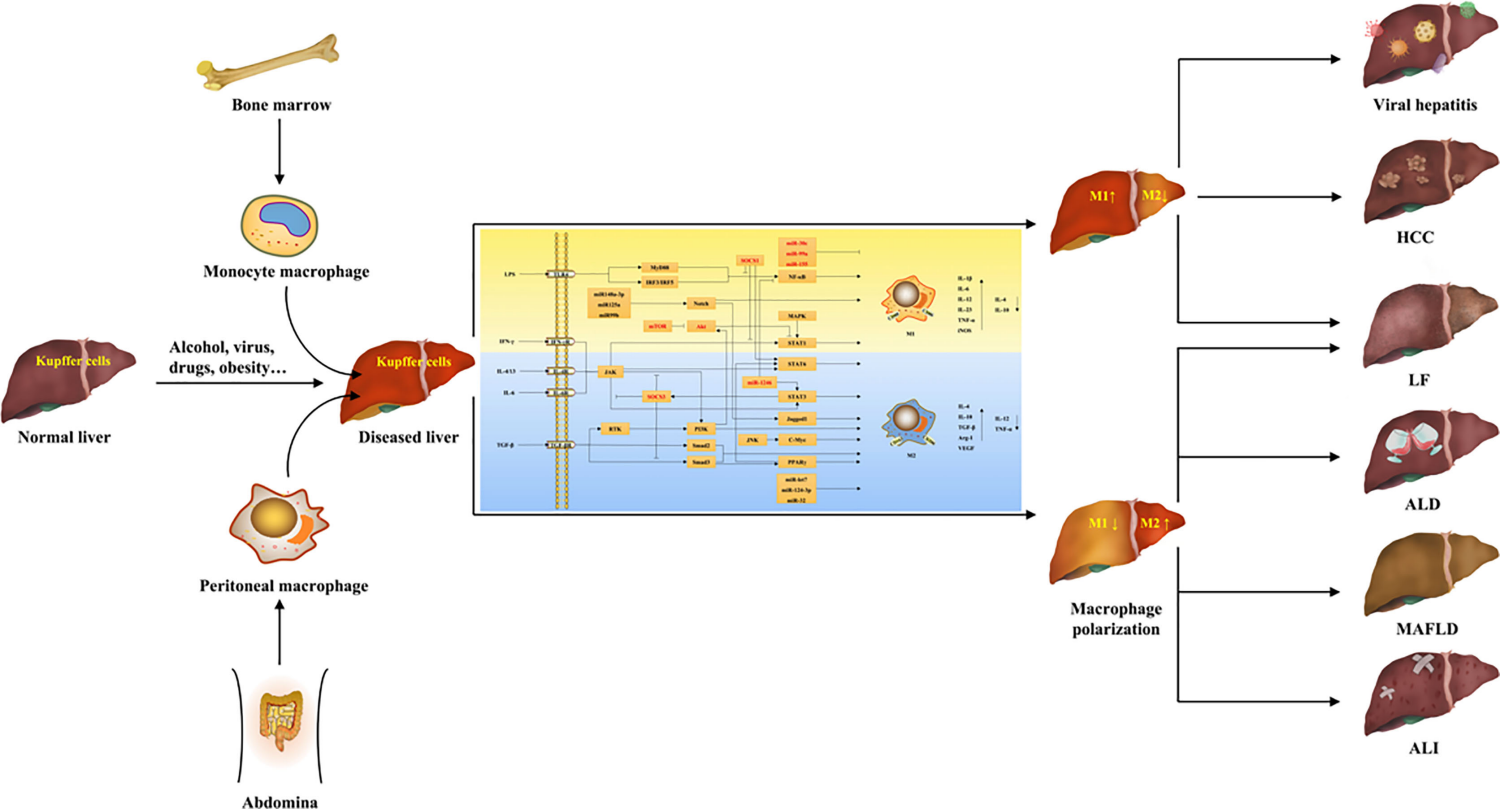


## 1 Introduction

The liver is an essential organ for maintaining normal life activities of the human body, because it not only regulates the metabolism of many nutrients and chemical drugs, but also has many functions such as synthesizing and decomposing proteins, regulating systemic blood volume, excluding body toxins, and regulating immunity (1). Liver diseases refer to hepatic pathological changes caused by a variety of pathogenic factors both inside and outside, which largely affect the normal physiological function of the human body. At present, numerous factors can trigger liver diseases, such as drugs, chemical agents, viral infection, excessive alcohol consumption, malnutrition, acid-base disorders, etc. (2–4). Based on the different etiologies and pathogenesis, liver diseases are classified as acute liver injury (ALI), viral hepatitis, alcoholic liver disease (ALD), metabolic-associated fatty liver disease (MAFLD), liver fibrosis (LF), cirrhosis, and hepatocellular carcinoma (HCC) (5). Due to the changes of living environment and the guaiac irregularity of life habits, the number of patients with liver diseases is increasing worldwide in recent years, which has gradually developed into a global public health problem. According to the epidemiological statistics, the number of global deaths caused by end-stage liver diseases such as viral hepatitis, cirrhosis and HCC is up to 2 million every year (6). Therefore, clarifying the pathogenesis of liver diseases and developing drugs for their targeted therapy are of great significance for the clinical treatment of liver diseases.

Macrophages are essential components of the innate immune system, and the activation of macrophages has been shown to be indispensable in several aspects, such as immune defense, inflammatory response, tissue remodeling, and homeostasis (7). Macrophages are distributed in nearly all tissues of the body, and are remarkably heterogeneous (8). In addition to the self-renewing resident macrophages originating in the yolk sac or embryonic hematopoietic stem cells, particularly under pathological conditions, macrophages of other origins are also continuously recruited to the tissues (8). For example, in liver tissues, in addition to Kupffer cells residing permanently within the hepatic sinuses, there are also abdomen-derived macrophages and bone marrow-derived monocyte macrophages (9). More importantly, macrophages have extreme plasticity, which can exhibit different activation states due to the changes of tissue microenvironment (10). Macrophages differentiate into different phenotypes under the stimulation of various factors, and exhibit different characteristics and effects, thus exerting different regulatory functions in the body’s physiological and pathological activities, which is also known as the polarizing effect of macrophages (10).

In recent years, a large body of literature has shown that macrophage polarization plays a crucial role in many pathophysiological processes, such as inflammation, tumor, tissue repair, and metabolism (11–13). Interestingly, these pathological processes are precisely also present in liver diseases, suggesting that macrophage polarization may be critically involved in the development and reversal of several liver diseases, such as fatty liver disease, hepatitis, fibrosis, and HCC (14–16). With the further study of macrophage polarization in liver diseases, targeting macrophage polarization to block or even reverse hepatic pathological changes has been considered as a potential strategy for the treatment of liver diseases (17). However, the origin of hepatic macrophages and the mechanism of macrophage polarization are complex, and their effects on different types of liver diseases and even on different stages of one liver disease are not the same (18). Therefore, the process of macrophage polarization and its role and mechanism on liver diseases need to be further studied and elucidated. By searching the online databases including PubMed, Web of Science, Google Scholar, and CNKI, the origin of hepatic macrophages and the diverse molecular mechanisms of macrophage polarization as well as its regulation in various liver diseases are summarized in this review. It is hoped to provide direction and basis for future research on the mechanism of macrophage polarization and on the treatment of liver diseases *via* regulating macrophage polarization.

## 2 The Origin of Hepatic Macrophages

Hepatic macrophages account for 90% of the total macrophages in the human body and are remarkably heterogeneous, including liver-resident macrophages and a variety of infiltrating macrophages (8, 17). Liver-resident macrophages, called Kupffer cells, generally exist in the hepatic sinuses and originate from yolk sac-derived specific progenitor cells having been seeded in liver tissue during embryogenesis, which can also be replenished by differentiation of bone marrow-derived monocytes (19, 20). Kupffer cells are self-renewed, quiescent and non-migratory in the liver, and have functions such as clearing pathogens, phagocytosing cellular debris, and regulating iron metabolism, which are important for maintaining liver homeostasis (20). In addition, the infiltrating macrophages include bone marrow/monocyte-derived macrophages, peritoneal macrophages, and splenic macrophages (9). Among them, bone marrow-derived monocyte macrophages are the main members of infiltrating macrophage and recruited after Kupffer cell and HSC activation, which are important contributors to the replenishment and regeneration after hepatic macrophage depletion, and have an important status in the pathological state of the liver (9). In addition to monocyte macrophages in the blood circulation, self-renewal macrophages in the peritoneal cavity also accumulate in subcapsular liver tissue when liver injury occurs and contribute to liver regeneration (17). Furthermore, the spleen has also been found to be the site of monocyte storage and distribution, and splenic macrophages are recruited to the liver during liver injury and have immunomodulatory effects (17). These macrophages have great plasticity (polarization) and usually exist with two main subsets. For example, peritoneal macrophages are divided into large peritoneal macrophages and small peritoneal macrophages (20). Interestingly, there are studies showing the presence of two monocyte macrophage subsets called ly6chigh and ly6clow in mice, and ly6chigh originates mainly from bone marrow, whereas ly6clow is derived from spleen (20). Collectively, in normal liver, Kupffer cells, known as sentinel cells of the liver, account for the majority of hepatic macrophages and are dominant, which mainly maintain liver homeostasis (20). When the liver is invaded by external factors to develop lesions, Kupffer cells first receive the signals to differentiate into different phenotypes to produce pro- or anti-inflammatory factors, and recruit a large number of other macrophages into the liver at the same time, which have similar plasticity and multiple functions as Kupffer cells, and play an important role in the progression and reversal of liver diseases (9, 17).

## 3 Macrophage Polarization

Macrophage polarization means that macrophages are activated under the stimulation of pathogenic microorganisms, inflammatory responses, cytokines, or some physicochemical factors, and differentiate into different phenotypes depending on the state and changes of the microenvironment (21). In the process of disease occurrence and regression, macrophage polarization appears to act as an intermediate process, which is activated by certain signals to generate distinct phenotypes first, thus playing a regulatory role by acting on multiple signaling pathways (22). The phenotypes, mechanisms, and functions of macrophage polarization are summarized in **Table 1**.

**Table 1:** The phenotypes, mechanisms and functions of macrophage polarization.

Macrophage phenotypes		Stimulus	Specific markers	Cytokines	Mechanisms	Functions	References
M1		LPS, IFN-γ, GM-CSF	CD80, CD86, CD16/32, MHCII, iNOS	IL-1β, IL-6, IL-12, IL-23, TNF-α, CXCL1~3, CXCL8~10, CCL2~5, CCL11	TLR4/NF-κB, IRF5, JAK/STAT1, Notch	Antigen presentation, Th1 immune reaction, proinflammation, pathogen elimination, anti-tumor	(10, 23–25)
M2	M2a	IL-4, IL-13	CD206, MHCII, IL-1R, Dectin-1	Arg1, IL-10, TGF-β, CCL17, CCL22	JAK/STAT6, c-Myc, IRF4	Anti-inflammation, wound healing, Th2 immune response, anaphylaxis, fibrosis	(26, 27)
	M2b	LPS, IC	CD206, MHCII, CD86	IL-10, IL-1β, IL-6, TNF-α, IL-12^low^	TLR4, Syk, PI3K	Immune regulation, pro-tumor, promoting infection	(26–28)
	M2c	IL-10, TGF-β, glucocorticoid	CD206, CD163	IL-10, TGF-β, Arg-1, CXCL13	JAK/STAT3, NF-κB, TGF-β/Smads	Phagocytosis, immunosuppression, tissue remodeling	(26, 28)
	M2d	TLR agonist, A_2A_R ligand	CD206	IL-10, VEGF, IL-12^low^, TNF-α^low^	TLR4, NF-κB	Pro-tumor, angiogenesis	(29, 30)
M4		CXCL4	MMP7^+^S100A8^+^, CD206, CD163^-/-^	TNF-α, CCL18	N/A	Proinflammation, low phagocytosis	(31)
Mox		QxPAPC	HO-1, Srxn1, Gclc, Gclm Txnrd1, Nurr1, Trb3, COX-2	IL-1β, VEGF	Nrf2, Keap1, TLR2	Low chemotaxis and phagocytosis	(26, 32)
M(Hb)		Hemoglobin	CD206, CD163	IL-10, IL-1R antagonist	PI3K/AKT, LXRα	Cholesterol loading resistance, ATP-binding cassette transporter up-regulation	(26, 33)

### 3.1 The Phenotypes of Macrophage Polarization

In general, the phenotypes of macrophage polarization can be divided into classically activated M1 and alternatively activated M2 (25). Nowadays, it is generally accepted that M1 macrophages are mainly induced by lipopolysaccharide (LPS) and interferon-γ (IFN-γ), whereas interleukin (IL)-4 and IL-13 can activate M2 macrophages (7). M1 macrophages are also known as pro-inflammatory macrophages because they can secrete a large number of pro-inflammatory cytokines, such as IL-1β, inducible nitric oxide synthase (iNOS), tumor necrosis factor-α (TNF-α) (10). Conversely, M2 macrophages are known as anti-inflammatory macrophages because of mainly producing anti-inflammatory factors, such as IL-10, transforming growth factor-β (TGF-β), arginase 1 (Arg1) (10). M1 macrophages mainly exert antigen-presenting function, and have pro-inflammatory, scavenging pathogenic microorganisms, and anti-tumor effects; while M2 macrophages have the biological functions of inhibiting inflammation, promoting tissue remodeling, preventing parasitic infection, as well as involving angiogenesis, immunity regulation, and tumor progression (23). Therefore, they usually exert opposite regulatory roles in the initiation and development of many diseases. Moreover, because M2 macrophages produce complex cytokines and have various functions, they can be further divided into M2a, M2b, M2c, and M2d subtypes (30).

**Figure 1:**
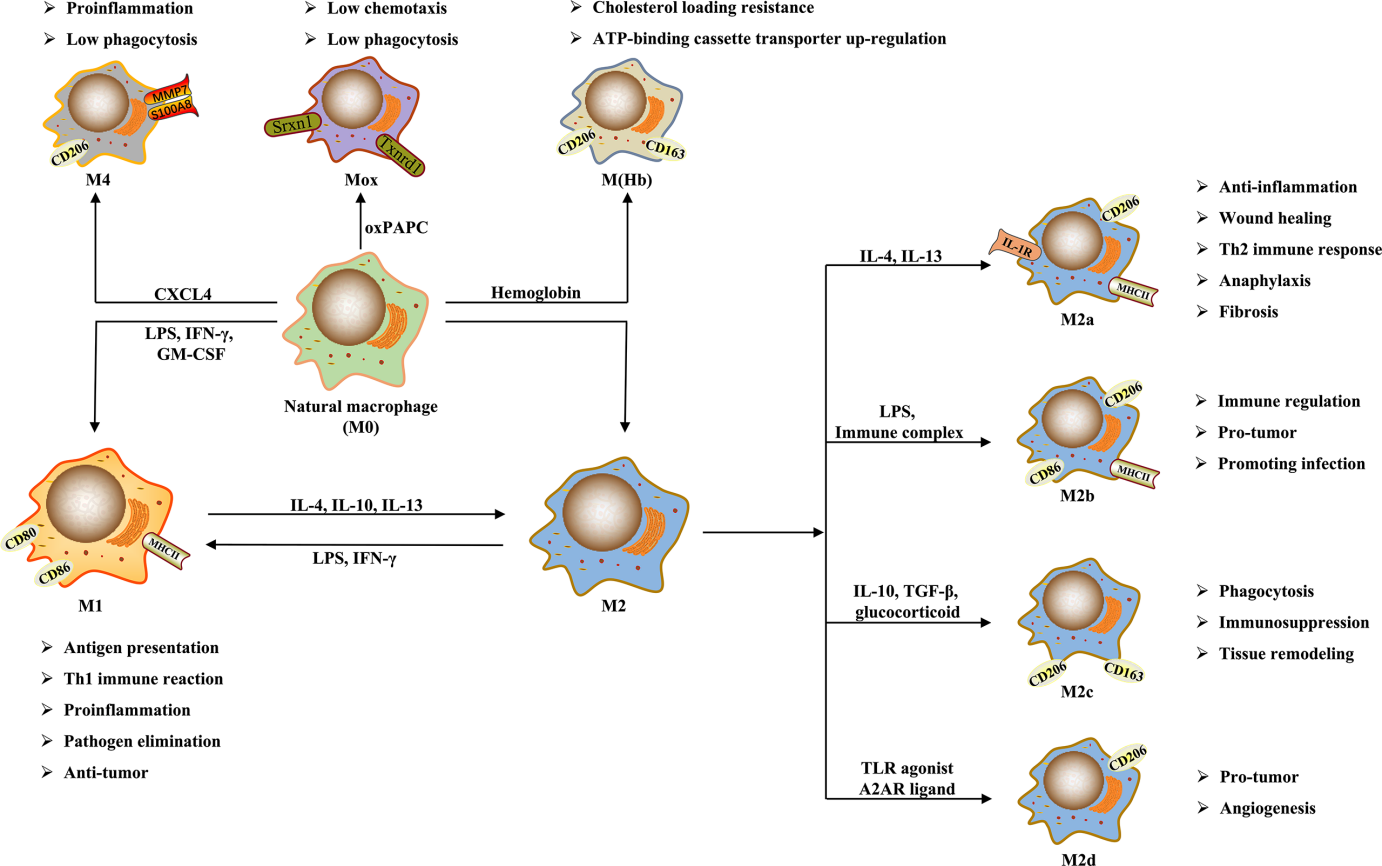
The phenotypes and functions of macrophage polarization.

Although M1 and M2 are the main macrophage phenotypes which are commonly studied and applied currently, the phenotypes of macrophage polarization are not restricted to them. The study by Erbel et al. (31) has shown that CXCL4 can induce a macrophage phenotype called M4, characterized by the co-expression of CD68, matrix metalloproteinase (MMP) 7, and the calcium binding protein S100A8, which has regulatory effects on diseases such as atherosclerosis. Moreover, in atherosclerosis, human hemoglobin can induce M(Hb) macrophage that highly expresses the heme scavenger receptor CD206 and CD163, and oxidized phospholipids in mice can induce Mox macrophage (26, 32). These macrophages have different morphological structures, gene expression, and biological functions from M1 and M2. Furthermore, Malyshev et al. (34) made a hypothesis that macrophages might achieve the interconversion of M1 and M2 by forming the M3 switching phenotype. The phenotypes and functions of macrophage polarization are shown in **Figure 1**.

### 3.2 The Mechanism of Macrophage Polarization

#### 3.2.1 TLR4/NF-κB Signaling Pathway

Toll like receptor (TLR) 4 is an innate immune receptor expressed on the surface of macrophages that can efficiently recognize pathogen-associated molecular patterns, and is the main receptor of LPS (35). LPS binds to TLR4 to activate nuclear factor-κB (NF-κB) through the myeloid differentiation factor 88 (MyD88)-dependent pathways or interferon regulatory factor (IRF) 3, thereby promoting the expression of inflammatory factors (36). Recently, a variety of drugs have been shown to inhibit M1 macrophage polarization by inhibiting the TLR4/NF-κB signaling pathway. For example, berberine could competitively inhibit the combination of TLR4 and MyD88 to inhibit the TLR4/MyD88/NF-κB signaling pathway, thus inhibiting M1 macrophage polarization (37). Similarly, quercetin downregulated the expression of NF-κB and IRF5, and then inhibited the activity of upstream TLR4/MyD88 to inhibit M1 polarization (38, 39). In addition, the chemical compounds NZ, meisoindigo, and others can inhibit M1 macrophage polarization, which is associated with the down-regulation of the TLR4/NF-κB signaling pathway (38, 40). These findings sufficiently indicate the critical role of TLR4/NF-κB signaling pathway in M1 macrophage polarization.

#### 3.2.2 JAK/STAT Signaling Pathway

The Janus kinase (JAK)/signal transducer and activator of transcription (STAT) pathway mainly mediates the signaling of cytokine receptors (41). IFN-γ binds to its receptor and activates JAK, thus inducing the phosphorylation of STAT1, which leads to the polarization of macrophages to M1 (42). Moreover, IFN-γ can enhance the sensitivity of macrophages to inflammatory mediators, and exert a synergistic effect by blocking the feedback inhibition to TLR signaling; meanwhile, NF-κB and mitogen-activated protein kinase (MAPK) can also enhance the transcriptional activity of JAK/STAT1 (42, 43). Research found that azithromycin promoted M2 polarization by inhibiting the transcription of STAT1 and NF-κB (44). In addition, IFN-γ can also promote the metabolic switch of M1 macrophages, which in turn enhances their cell viability and pro-inflammatory activity through the JAK/STAT1 pathway (45). JAK/STAT6 is an important pathway by which IL-4 inhibits M1 and induces M2 polarization (46). For example, curcumin up-regulated STAT6 expression by secreting IL-4 and IL-13, thereby inducing M0 and M1 macrophages to polarize to M2 (47).

In addition, STAT3 is important for M2 macrophage polarization. Studies have shown that the inhibition of IL-6/STAT3 and JAK3/STAT3 signaling pathways results in the polarization of macrophages from M2 to M1 phenotype (48, 49). Suppressor of cytokine signaling (SOCS) is a feedback inhibitor of JAK/STAT signaling. It was found that the deficiency of SOCS1 and SOCS3 promoted M1 macrophage polarization by activating the JAK1/STAT1 signaling pathway (50, 51). The study by Yu et al. (52) further showed that increased phosphorylation of STAT3 could feedback inhibit the expression of STAT1 by upregulating the expression of SOCS3, thereby inhibiting macrophage polarization mediated by the JAK/STAT1 signaling pathway. In summary, M1 macrophage polarization is closely related to the phosphorylation of STAT1, whereas M2 polarization mainly depends on the increased expression of STAT3, STAT6, and SOCS.

#### 3.2.3 TGF-β/Smads Signaling Pathway

TGF-β acts on type II receptors first, and then binds to type I receptors to form a receptor complex, which leads to the phosphorylation of type I receptor domain, thus regulating the expression of the related genes by activating their downstream signaling molecules (Smad2 and Smad3) (53). The study by Wang et al. (54) found that growth differentiation factor 3 from the TGF-β superfamily suppressed M1 and promoted M2 polarization by promoting the phosphorylation of Smad2 and Smad3. Similarly, there have been multiple studies showing the role of TGF-β/Smads signaling pathway in promoting M2 macrophage polarization. For example, quercetin was found to inhibit M2 polarization through inhibiting TGF-β1-smad2/3 pathway (39). In addition, both TGF-β and Smads signaling can individually mediate macrophage polarization. For example, under hypoxia, TGF-β expression was upregulated, which might increase M2 polarization through the receptor tyrosine kinase/phosphatidylinositol-3-kinase (PI3K) pathway (55). Chen et al. (56) experimentally demonstrated that Smad3 could be directly activated by macrophage phagocytosis independently of TGF-β, which might promote macrophage polarization toward the anti-inflammatory phenotype *via* peroxisome proliferators-activated receptors (PPARs).

#### 3.2.4 PPARγ Signaling Pathway

PPARγ is an important transcription factor for cell differentiation and has many functions, such as regulating glucose and lipid metabolism, anti-inflammation, reducing oxidative stress, and so on (57). PPARγ usually regulates the polarization of macrophages by interacting with other signaling pathways. The study by Luo et al. (58) showed that PPARγ interacted with NF-κB to regulate the balance of M1/M2 macrophages. Gao et al. (47) found PPARγ was involved in the process of M2 macrophage polarization induced by IL-4/IL-13. This suggests that PPARγ plays an important role in M2 macrophage polarization. For example, HuoxueTongfu formula activated PPARγ to up-regulate the expression of SOCS1/STAT6 signaling pathway and inhibit SOCS3/JAK2/STAT1 pathway, thereby promoting M2 polarization (59). In addition, insulin and α-ketoglutarate can also increase the expression of PPARγ signaling to promote M2 polarization, thus slowing down the development of various inflammatory diseases (60, 61).

#### 3.2.5 MicroRNAs

According to the current research, the role of microRNAs (miRNAs) on macrophage polarization has been gradually highlighted, mainly relying on their regulation of other signaling pathways. For example, miR-221-3p promotes M2 macrophage polarization toward M1 phenotype by inhibiting JAK3/STAT3 signaling pathway (49). MiR-1246 induces M2 polarization by targeting TERF2IP to activate STAT3 and inhibit NF-κB (62). In recent years, exosomes have been extensively studied and applied, and have been shown to be important carriers of miRNA signaling (63). Adipocyte-derived exosomes carry miR-34a, which can suppress Krüppel like factor 4 expression and inhibit M2 polarization (64). Conversely, mesenchymal stem cell (MSC)-derived exosomal miR-124-3p can promote M2 macrophage polarization (65). Meanwhile, macrophage-derived exosomes can also exert their biological effects *via* miRNAs. M2 macrophage-derived exosomes led to the exacerbation of pancreatic ductal adenocarcinoma through the inhibitory effect of miR-501-3p on TGFBR3, and down-regulated TXNIP expression as well as inhibited the TLR4/NF-κB/NOD-like receptor protein 3 (NLRP3) inflammasome signaling pathway *via* miR-148a, thus attenuating myocardial ischemia/reperfusion injury (66, 67). Moreover, miR-30c, miR-99a and miR-155 have all been shown to inhibit M1 macrophage polarization, while miR-let7 and miR-32 contribute to M2 polarization (68–72).

#### 3.2.6 Notch Signaling Pathway

Notch signaling pathway includes a series of highly conserved surface receptors, and is involved in cell proliferation and apoptosis, affecting the development of various biological organs and tissues (73). A study has found that M1 macrophages have a marked increase in Notch1 receptor expression, and Notch1 receptor inhibition leads to decreased M1 polarization and increased M2 polarization (74). In recent years, much literature has reported that many drugs can regulate macrophage polarization by targeting the Notch signaling pathway. For example, astragalus polysaccharide activates the Notch signaling pathway to induce M1 polarization (75). Capsaicin inhibits M1 polarization by inhibiting the Notch signaling pathway (76). In addition, Zheng et al. (77) found that Notch1/Jagged1 signaling inhibition could suppress schistosome infection-induced M2 polarization. Meanwhile, the study by Tao et al. (78) also showed that Linc00514 promoted M2 polarization through STAT3 and Notch/Jagged1 signaling pathway, leading to the development of breast cancer. Therefore, these suggest that the Notch/Jagged1 pathway may be a therapeutic target for some diseases associated with M2 macrophage polarization.

Interestingly, the current studies have shown that Notch signaling regulating macrophage polarization is closely associated with miRNAs. Li et al. (79) found that the mechanism by which Notch signaling promotes M1 polarization involved increased expression of miR-125a/miR-99b. Similarly, miR-148a-3p was shown to be a mediator by which Notch promotes M1 polarization (80). Moreover, adipose stem cell-derived extracellular vesicles (EVs) could inhibit the Notch signaling pathway and M1 polarization to exert anti-inflammatory effects, which was associated with decreased expression of miR148a-3p (81).

#### 3.2.7 Other Mechanisms

In addition to the pathways described above, multiple other signals and targets have been included in the underlying mechanisms of macrophage polarization. Mammalian target of rapamycin signaling pathway was shown to be involved in regulating M1/M2 polarization, mainly relying on the feedback effect between mechanistic target of rapamycin (mTOR) complex 1 and protein kinase B (Akt) signaling (82). Insulin suppressed NF-κB and STAT1 expression through the PI3K/Akt signaling pathway, thus reducing pro-inflammatory M1 macrophage activation (60). The study by Zhang et al. (83) showed that MCP-induced protein 1 promoted macrophage polarization from M1 to M2 by inhibiting the JNK/c-Myc signaling pathway. ω-alkynyl arachidonic acid promoted M2 polarization by regulating the crosstalk of pyruvate kinase M2, hypoxia inducible factor-1 α (HIF-1α) and iNOS, thus contributing to the attenuation of the inflammatory responses in acute myocardial infarction (84). Furthermore, Gu et al. (85) found that the effects of the N6 methyladenosine demethylase FTO on macrophage polarization were complex. On the one hand, FTO could mediate the phosphorylation of IKKβ, IκBα, and p65 to activate NF-κB signaling pathway and up-regulate STAT1 expression, thereby inducing M1 polarization (85). On the other hand, FTO deficiency not only inhibited M1 polarization, but also inhibited M2 polarization, which was related to the down-regulation of STAT6 and PPARγ (85).

**Figure 2:**
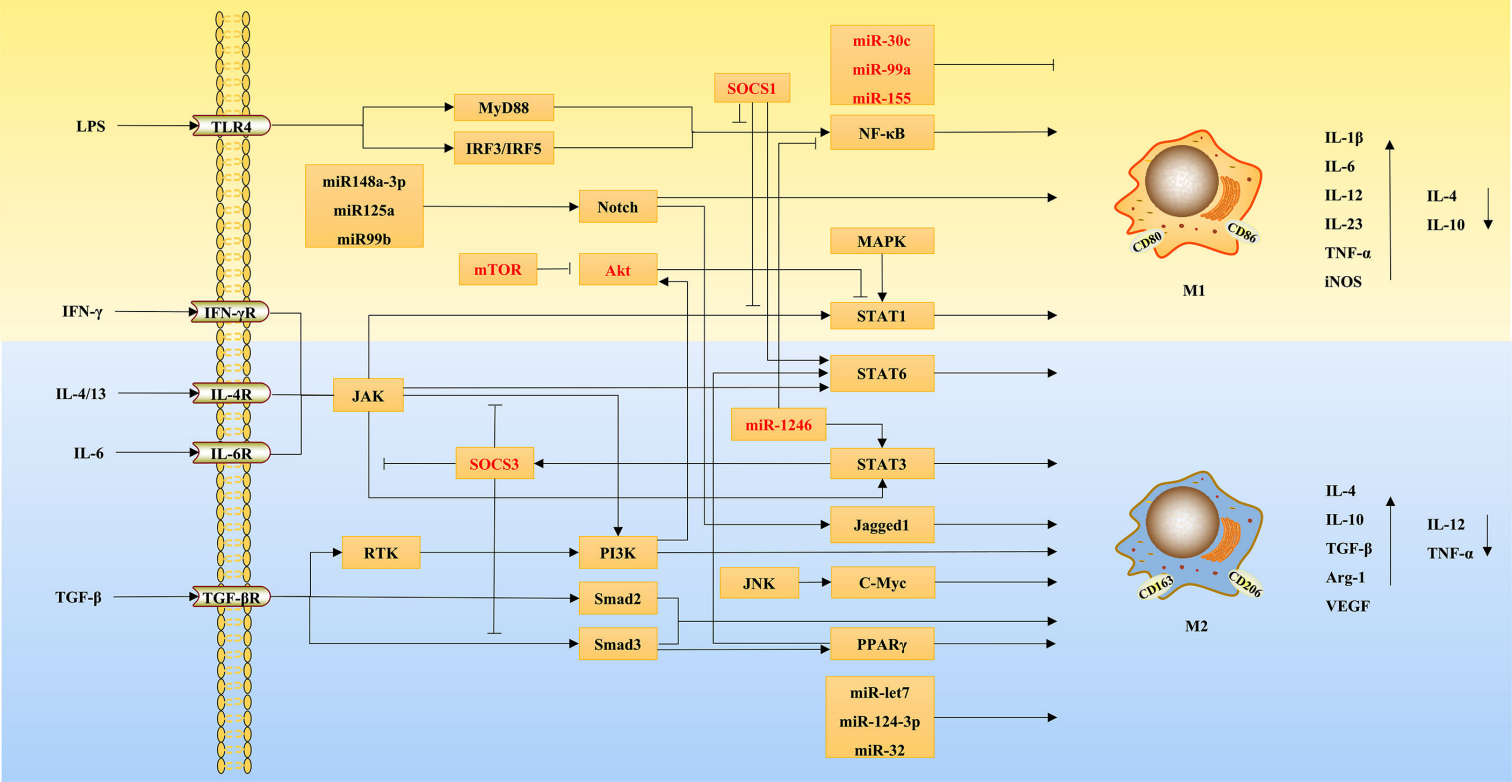
The mechanisms of macrophage polarization.

In fact, the mechanism associated with macrophage polarization is very extensive, and the induction of macrophage polarization by most factors involves the co-regulation of multiple signaling pathways, which is perhaps an important factor for macrophage polarization to have multiple roles in liver diseases. The detailed mechanisms of macrophage polarization are shown in **Figure 2**.

## 4 The Role of Macrophage Polarization in Liver Diseases

### 4.1 Acute Liver Injury

ALI is the acute hepatic inflammation and hepatocyte necrosis caused by endotoxin, certain drugs and their metabolites, or other physicochemical factors, which may cause liver dysfunction or even acute liver failure (86). Empirically, LPS, D-galactosamine (D-GalN), thioacetamide (TAA), and acetaminophen (APAP) can cause ALI and have mostly been used to establish experimental ALI models (87, 88). Through a study in the TAA-treated rats, Golbar et al. (87) demonstrated that the depletion of hepatic macrophages obviously aggravated liver injury. Zigmond et al. (89) further found that the macrophages of different origins differentiated into different subpopulations and had different functions in the APAP-induced ALI mouse model. In addition, the study by Rahman et al. (90) showed that M1 and M2 macrophages together contributed to D-GalN-induced ALI in rats and interconverted during the lesion progression. These findings provided ample evidence that macrophages played an important role in ALI, and indicated that it was possible to control the disease progression by regulating macrophage polarization.

JAK/STAT1 and TLR4/NF-κB pathways are important mechanisms of M1 macrophage polarization, while STAT6 is mainly related to M2 (45, 46, 91). Through the *in vivo* and *in vitro* experiments, Xie et al. (92) demonstrated that protein interacting with C kinase 1 up-regulated the expression of STAT6 and p38α as well as inhibited the NF-κB signaling pathway, promoting M2 and inhibiting M1 polarization, which reduced the liver injury. MSC-derived prostaglandin E2 acted on EP4 receptor, and then alleviated hepatic inflammation by reducing the release of inflammatory factors as well as promoted M2 polarization by up-regulating the expression of STAT6 and mTOR signaling, thus alleviating ALI (93). Cannabinoid receptor 2 can down-regulate the expression of TLR4 signaling *via* miR145 and promote macrophage polarization from M1 to M2, thus playing a protective role in mice with acute liver failure (94). Conversely, Wang et al. (95) found that hyperglycemia promoted M1 and inhibited M2 polarization by up-regulating STAT1 and down-regulating STAT6 expression, and aggravated APAP-induced ALI in mice through the PI3K/Akt signaling pathway. Similarly, SMA/CORM2, a CO donor designed by Song et al. (96) for colitis, attenuated liver injury by reducing oxidative stress and modulating M1/M2 polarization, which was associated with the down-regulation of HIF-1α expression and the activation of the PI3K/Akt/mTOR signaling pathway. However, Gong et al. (97) conducted *in vivo* and *in vitro* experiments and demonstrated that the mechanism by which phenylenediamine analogue FC-99 attenuated liver injury was mainly through the M2 macrophage polarization mediated by PPAR-γ rather than STAT6.

CCL5 can act on CCR1/CCR5 receptor and up-regulate the expression of MAPK and NF-κB signaling pathways, which promotes M1 and inhibits M2 macrophage polarization (98). Peng et al. (99) and Liu et al. (100) demonstrated that the p300/CBP inhibitor and p38α deletion could contribute to liver injury amelioration by reducing CCL5 expression and inhibiting NF-κB signaling to regulate macrophage polarization []. In addition, Tsuji et al. (101) demonstrated the close relationship between M1/M2 macrophage polarization and autophagy by a study on the pathogenesis of ALI induced by APAP in rats. Further research by Zhou et al. (102) and Hua et al. (103) showed that both spermine and human amniotic mesenchymal stromal cells could inhibit M1 and promote M2 polarization by promoting autophagy, thus alleviating liver injury in different ALI mouse models.

M1 macrophages aggravate liver tissue injury because of promoting inflammatory responses; conversely, M2 macrophages can attenuate liver injury through their effects of anti-inflammation and tissue repairing (23). Therefore, in theory, the increase of M2 macrophage polarization or the inhibition of M1 polarization is good for ALI alleviation. In fact, the promoting or slowing effects of most internal and external factors on ALI are achieved by regulating macrophage polarization, as shown in **Table 2**.

**Table 2:** The role and mechanisms of macrophage polarization in ALI.

Regulation Factor	Research objects	Macrophage polarization	Mechanisms	Year and Country	Reference
**Alleviating ALI**					
Cannabinoid Receptor 2	Mice, cells	M1→M2	TLR4 signaling↓; miR-145↓	2015, America	(94)
Protein interacting with C kinase 1	Mice, cells	M1↓; M2↑	NF-κB signaling↓; STAT6 signaling↑	2016, China	(92)
P38α deficiency	Mice, cells	M1→M2	CCL2, CCL5↓; p38α-CREB-C/EBPβ↓	2017, China	(100)
Spermine	Mice, cells	M1↓; M2↑	STAT1↓, STAT6↑; ATG5↑	2018, China	(102)
Homeobox Containing 1	Cells, mice	M1↓	NF-κB signaling↓; MHCII↓	2018, China	(104)
Benzenediamine Analog FC-99	Mice, cells	M1↓; M2↑	PPAR-γ signaling↑	2019, China	(97)
p300/CBP inhibitor A-485	Mice, cells	M1↓	H3K27ac/H3K18ac↓; NF-κB, MAPK, NLRP3 signaling pathway↓	2019, China	(99)
Human amniotic mesenchymal stromal cells	Mice, cells	M1↓; M2↑	LC3B-II↑	2019, China	(103)
Carbon monoxide	Mice, cells	M1↓; M2↑	HIF-1α↓; PI3k/Akt/mTOR signaling↑	2021, China	(96)
Msc-secreted prostaglandin E_2_	Mice, cells	M2↑	STAT6 and mTOR signaling↑	2021, China	(93)
**Aggravating ALI**					
Hyperglycemia	Mice, cells	M1↑; M2↓	STAT1↑, STAT6↓; AMPK↓, PI3K/AKT pathway↑	2019, China	(95)
CCL5	Patient samples, mice	M2 ↓	MAPK and NF-κB signaling pathway↑	2020, China	(98)

### 4.2 Viral Hepatitis

Viral hepatitis is a class of infectious diseases caused by hepatitis virus with hepatocyte degeneration, necrosis and apoptosis as the main lesions, including five types: A, B, C, D, and E (105). It is very likely to form chronic hepatitis after hepatitis virus infection, and then progress to liver fibrosis, ultimately leading to cirrhosis and even HCC (105). Currently, inhibiting the replication and spread of hepatitis virus by regulating the body’s immune system is the key to the treatment of viral hepatitis (106). Macrophage, as an important immune cell, is considered to be an important player in the development and resolution of chronic viral hepatitis (107). When viral infection occurs in the liver, Kupffer cells will recognize danger signals first, and trigger the recruitment of circulating monocytes to the liver and subsequent differentiation to macrophages, together exerting immunoregulatory function as well as having pathogen elimination and anti-viral effects (18).

Hepatitis B, mainly caused by hepatitis B virus (HBV) infection, is one of the leading causes of chronic hepatitis worldwide (105). It is generally believed that HBV-related liver damage is associated with the killing of infected-hepatocytes by CD8^+^ T lymphocytes. Meanwhile, the anti-viral function of CD8^+^ T lymphocytes is regulated by hepatic regulatory T cells. Dai et al. (108) found that CD206-positive macrophages were predominant in HBV-infected mice and produced amphiregulin to up-regulate the immunosuppressive activity of regulatory T cells, impairing the anti-viral effect of CD8^+^ T cells, which was associated with rapamycin signaling activation. Similarly, Yi et al. (109) found that hepatitis B core antigen significantly inhibited M2 polarization and the production of anti-inflammatory factors by activating the TLR2-NF-κB signaling pathway, which exerted therapeutic potential against chronic hepatitis B. In addition, studies have shown that miRNAs are also involved in regulating the occurrence and resolution of hepatitis B. Zhao et al. (110) have proved that HBV-encoded miR-3 can promote M1 macrophage polarization to exert anti-HBV effects, which may be through suppressing SOCS5 expression to activate the JAK/STAT signaling pathway (110).

Hepatitis C is a viral hepatitis caused by hepatitis C virus (HCV) infection with a worldwide prevalence of approximately 3% and an increasing trend (111). G. Dultz et al. (112) found that the serum level of soluble CD163, a marker of M2 macrophage activation, was significantly increased in HCV-infected patients, but decreased after the anti-viral therapy, confirming the crucial role of M2 polarization in the progression of hepatitis C. Moreover, a recent study showed that the M1 macrophages from the livers of HCV-infected patients exhibited M2 phenotypic features, and the M2 macrophages exhibited M1 phenotypic features (113). Similarly, a study by Saha et al. (114) found that HCV-infected HCC cells induced monocytes to differentiate into macrophages and polarize to M2 phenotype. Moreover, a previous study showed that exogenous HCV core protein promoted macrophages to secrete pro-inflammatory and anti-inflammatory factors, and mediated the pro-proliferative effect of macrophages on human normal hepatocyte line LO2, which was accompanied by increased expression of M2-associated STAT3 and CD206 (115). Subsequently, further research showed that HCV core protein inhibited M1 and M2 macrophage polarization through the TLR2/STATs pathway, and impaired their phagocytic activity and functions (116).

Vaccine is an important means to prevent and control viral hepatitis, and the development of an effective vaccine against HCV infection is of great importance. Ohtsuki et al. (117) showed that the number of M2 macrophages in the liver of HCV-infected mice was significantly increased, accompanied by high expression of IL-6 and TNF-α, whereas the recombinant vaccinia virus expressed HCV nonstructural protein rVV-N25 and inhibited the number and activation of M2 macrophages, thus preventing the development of chronic hepatitis. Taken together, M2 macrophages have an important role during HBV and HCV infection, and are mostly accompanied by a complex cytokine profile, not only including increased M2-related anti-inflammatory cytokines but also involving M1. These results suggest that M2 macrophage polarization inhibition and M1-related inflammatory factor secretion may contribute to the inhibition of virus replication and infection, thus alleviating viral hepatitis and inhibiting related fibrosis. The role and mechanisms of macrophage polarization in viral hepatitis are shown in **Table 3**.

**Table 3:** The role and mechanisms of macrophage polarization in viral hepatitis.

Regulation Factor	Research objects	Macrophage polarization	Mechanisms	Year and Country	Reference
**Alleviating hepatotropic virus infection**					
Scavenger receptor-AI	Mice, cells	M2↑	MerTK↑, mTOR pathway↓	2017, America	(118)
**Alleviating HBV infection**					
Hepatitis B Core Antigen	Patient samples, cells	M2↓	TLR2/NF-κB pathway↑; STAT6↓	2020, China	(109)
HBV-miR-3	Patient samples, cells	M1↑	SOCS5↓; JAK/STA T signaling pathway↑	2020, China	(110)
**Aggravating HCV infection**					
HCV antigens	Patient samples, cells	M1↓	A20/A20-binding inhibitor of NF-κB binding protein↑; NF-κB signaling↓	2015, China	(119)
HCV core protein	Patient samples, cells	M1↓; M2↓	TLR2 signaling↑; STAT1↓, STAT3↓	2016, China	(116)
HCV single-stranded RNA	Patient samples, cells	M2↑	TLR7/8 signaling↑	2017, America	(120)
HCV E2 envelope glycoprotein	Cells	M2↑	STAT1↓; STAT3↑	2019, America	(121)

### 4.3 Alcoholic Liver Disease

ALD mainly refers to the hepatic inflammatory responses induced directly or indirectly by ethanol and its derivatives during their metabolism, which is the result of the interaction of various factors including oxidative stress, gut-derived endotoxin, inflammatory mediators, and nutritional imbalance (122). In particular, the activation of Kupffer cells by endotoxins due to impaired intestinal barrier function plays an important role in the initiation and progression of ALD (123). When ALD occurs, Kupffer cell activation plays a central role; meanwhile, monocyte macrophages are recruited to the liver and polarized to M1 or M2 phenotype according to the state of the liver microenvironment (19). Voican et al. (124) found that macrophage infiltration in subcutaneous adipose tissue was reduced, and M2 macrophage polarization was increased in the ALD patients with alcohol withdrawal. Moreover, M2 macrophages were shown to induce hepatocyte senescence *via* IL-6 and resist alcohol-induced hepatocyte apoptosis and hepatic steatosis (125). Therefore, the pathogenesis of ALD involves macrophage polarization, and M2 macrophages seem to be beneficial for ALD resolution.

Ethanol can significantly induce the expression of telomerase reverse transcriptase *in vitro* and *in vivo*, thereby promoting M1 macrophage polarization through the NF-κB signaling pathway, which has an important role in the pathogenesis of ALD (126). Cannabinoid CB2 receptor activation attenuates alcohol-induced hepatic steatosis and inflammation by inhibiting M1 polarization of Kupffer cells, and partially by promoting M2 polarization (127). β-caryophyllene, a food additive, can reduce M1 activation of Kupffer cells and contribute to the amelioration of ALD, which is partially dependent on CB2 (127). TLRs are widely expressed in ALD. Research has shown that the inhibition of TLR2 expression and the promotion of TLR3 expression in Kupffer cells can activate STAT3 and produce IL-10, which is beneficial to promote M1 to M2 polarization and then alleviate ALD (128). In addition, Fas, an apoptosis-related receptor, has a dual role in the development of ALD. On the one hand, Fas receptor favored early ethanol-induced M1 macrophage polarization and inflammatory responses (129). On the other hand, it reduced TGF-β production by inhibiting M2 polarization, and subsequently inhibited the pro-fibrotic responses in chronic ALD (129). Moreover, in the ethanol-induced *in vitro* and *in vivo* models, the overexpression of brain and muscle Arnt-like protein-1 could inhibit M1 and promote M2 polarization through glycolysis pathway, thus alleviating ALD (130).

Saha et al. (131) conducted in-depth studies on ALD, and found that both M1 and M2 polarization of hepatic macrophages were increased in the mouse model of chronic alcohol exposure, which was significantly associated with the modulation of Krüppel-like factor 4 expression by ethanol and its metabolite acetaldehyde. EVs carry a large number of proteins and miRNAs, which have been shown to be important mediators of intercellular signaling (132). Saha et al. (133) further found that the mice with ALD had an increased number of EVs, which carried specific proteins such as Hsp90 and increased the number of M1 macrophages as well as the infiltration of monocytes/macrophages. In addition, their findings showed that miR-27a from alcohol-exposed monocyte-derived EVs could induce naïve monocytes to differentiate into M2 macrophages (134). During alcoholic hepatitis, the regulation of miRNAs associated with macrophage polarization is disordered, and hepatic macrophages become less sensitive to LPS and undergo M1/M2 hyperpolarization (135).

Currently, endoplasmic reticulum stress has been shown to contribute to M2 macrophage polarization (136). Park et al. (137) found that NOGO-B, a protein that maintains the structure of endoplasmic reticulum, could promote M1 polarization of Kupffer cells and then aggravate alcoholic liver injury. Conversely, its absence contributed to increased endoplasmic reticulum stress and M2 polarization (137). More importantly, according to recent studies, pharmacological intervention targeting M2 macrophage polarization may be an effective approach for the treatment of ALD during the early inflammatory phase. For example, inulin can inhibit short chain fatty acid-induced M1 polarization, and promote M2 polarization, attenuating the inflammation of ALD mice (138). Furthermore, the study by Patel et al. (139) has shown that probiotics and metformin, as well as their combination, can promote M2 polarization and inhibit M1 polarization, contributing to the alleviation of alcoholic liver injury. The role and mechanisms of macrophage polarization in ALD are shown in **Table 4**.

**Table 4:** The role and mechanisms of macrophage polarization in ALD.

Regulation Factor	Research Objects	Macrophage polarization	Mechanisms	Year and Country	Reference
**Alleviating ALD**					
Cannabinoid CB_2_ receptor	Mice, cells	M1↓	HO-1↑; NF-κB signaling↓	2011, France	(127)
EV-miR-27a	Patient samples, cells	M2↑	IL-10, TGF-β↑	2016, America	(134)
β-caryophyllene	Mice	M1↓	Cannabinoid 2 receptors↑; PPARα↑	2018, America	(140)
β-hydroxybutyrate	Patient samples, mice, cells	M2↑	Hcar2-cAMP pathway↑	2018, America	(141)
Salidroside	Cells	M1↓	Notch-Hes signaling pathway↓; NF-κB↓	2019, China	(142)
Inulin	Mice, cells	M1↓; M2↑	Short chain fatty acids↑; TLR4-MyD88-NF-κB pathway↓	2020, China	(138)
Brain and Muscle Arnt-Like Protein-1	Mice, cells	M1↓; M2↑	S100A9 protein↑; glycolytic pathway↓	2021, China	(130)
Metformin and Probiotics	Cells, rats	M1↓; M2↑	MAPK/Nrf-2/HO-1 signaling pathway↑	2021, India	(139)
**Aggravating ALD**					
Telomerase reverse transcriptase	Mice, cells	M1↑	NF-κB pathway↑	2016, China	(126)
MiR-155	Mice, cells	M1↑; M2↓	PPARγ↓; C/EBPβ↓	2016, America	(143)
Nogo-B	Patient samples, mice, cells	M1↑; M2↓	C/EBP homologous protein↓; Endoplasmic reticulum stress↓	2017, America	(137)
EV-Heat shock protein 90	Mice, cells	M1↑; M2↓	IκB kinase↑, TLR4/NF–κB pathway↑	2018, America	(133)

### 4.4 Metabolic-Associated Fatty Liver Disease

MAFLD is a clinicopathological syndrome closely related to obesity, inflammation, and insulin resistance, which is mainly characterized by excessive lipid deposition in hepatocytes (144, 145). It was found that in the MAFLD mouse model induced by fructose rich and high-fat diet (HFD), the expression of M1 macrophage-related genes and signal pathways in the liver was increased significantly, while the expression of M2 markers was decreased (146, 147). Further research found that M1 macrophages could induce p62-positive inclusion body accumulation and chronic inflammation in the liver, thus leading to the exacerbation of MAFLD patients (148). In addition, Yoshii et al. (149) first used micromini pigs as animal models to explore the phenotypic changes of macrophages in HFD-induced MAFLD. The results showed that hepatic fat accumulation induced macrophage accumulation, and M2 macrophages were increased in the early stage of MAFLD, whereas M1 macrophages were increased in the late stage (149). These findings fully indicate that the occurrence and development of MAFLD are closely related to the M1/M2 polarization of macrophages, and regulating macrophage polarization may be an important strategy for the treatment of MAFLD.

Macrophage polarization involves various mechanisms, and similarly, the pathogenesis of MAFLD also includes various signaling molecules (24, 150). Targeting these signaling molecules to modulate macrophage polarization may be a potential avenue against MAFLD. For example, the activation of nuclear factor like 2 has been shown to improve obesity and insulin resistance in mice. Nagata et al. (151) showed that Glucoraphanin, a stable precursor of the nuclear factor like 2 inducer sulforaphane, reduced M1 macrophage activation and increased the number of M2 macrophages, which alleviated HFD-induced MAFLD. In addition, PPARγ is also a key pathway for M2 macrophage polarization. A study found that the up-regulation of PPARγ could promote Kupffer cells to convert from M1 to M2 phenotype in HFD-fed mice (58). In addition, long noncoding RNAs (lncRNAs) have also been found to be widely involved in MAFLD. For example, lncRNA SNHG20 silencing could attenuate inflammatory responses in MAFLD by inhibiting M1 polarization (152). However, its overexpression up-regulated STAT6, thus promoting M2 polarization and accelerating the progression of MAFLD to HCC (152). Similarly, diverse intestinal flora also promotes or reverses MAFLD/nonalcoholic steatohepatitis (NASH) by inducing macrophage polarization, such as Escherichia coli, Lactobacillus paracasei, etc. (153, 154).

Diabetic patients are usually accompanied by MAFLD, and many anti-diabetic drugs can improve MAFLD by inducing macrophage polarization. For example, rosiglitazone can attenuate hepatic steatosis and insulin resistance in MAFLD mice by promoting M2 and reducing M1 polarization (155). The mechanism involves the down-regulation of NF-κB signaling that interacts with PPARγ (155). In addition, liraglutide, a first-line drug for the treatment of type 2 diabetes, can induce anti-inflammatory M2 polarization of Kupffer cells *via* the cyclic adenosine monophosphate (cAMP)/protein kinase A (PKA)/STAT3 signaling pathway, and attenuate HFD-induced inflammation, which improves MAFLD (156). Similarly, saxagliptin, a novel anti-diabetic drug, was shown to promote M2 polarization by activating CaMKKβ/AMP-activated protein kinase (AMPK) signaling pathway and inhibit M1 polarization by decreasing NF-κB activity, which alleviated inflammatory responses in diabetic rats, thus ameliorating MAFLD (157). It is worth mentioning that metformin is also a common drug used in the clinical treatment of type 2 diabetes (158). Zamani-Garmsiri et al. (159, 160) found that, in HFD-fed mice, metformin combined with genistein or chlorogenic acid significantly reduced macrophage infiltration in the liver and induced macrophage polarization to M2 phenotype, thereby attenuating hepatic inflammation and MAFLD. The mechanism was associated with the inhibition of the NF-κB signaling pathway and the increase of AMPK expression (159, 160).

NASH, the hepatocyte inflammation based on hepatocyte steatosis, is a more severe stage of MAFLD (161). Through *in vitro* and *in vivo* experiments, Zhong et al. (162) demonstrated that honokiol promoted M2 polarization by activating PPARγ signaling, and attenuated high-cholesterol and high-fat diet-induced NASH. Similarly, cactus seed extract could attenuate hepatic steatosis and NASH in mice by modulating macrophage polarization (163). The mechanism might be related to the inhibition of the TLR4/NF-κB pathway and the promotion of PPARα expression (163). In addition, Yao et al. (164) found that myricetin suppressed M1 and promoted M2 polarization by inhibiting the TREM-1-TLR2/4-MyD88 signaling pathway and the phosphorylation of STAT3, thus attenuating the NASH induced by choline-deficient, L-amino acid-defined, and high-fat diet. β-cryptoxanthin and astaxanthin, two carotenoids with anti-oxidant effects, could inhibit M1 macrophage polarization to improve insulin resistance and NASH induced by a high-cholesterol and high-fat diet in mice (165, 166). Tyrosine kinase is important for NF-κB activation (167). Dasatinib is a tyrosine kinase inhibitor that prevents NF-κB activation, which can inhibit M1 and induce M2 polarization to alleviate western diet-induced NASH in mice (167). The role and mechanisms of macrophage polarization in MAFLD/NASH are shown in **Table 5**.

**Table 5:** The role and mechanisms of macrophage polarization in MAFLD/NASH.

Regulation Factor	Research Objects	Macrophage polarization	Mechanisms	Year and Country	Reference
**Alleviating MAFLD/NASH**					
β-Cryptoxanthin	Mice, cells	M1↓; M2↑	JNK, p38 MAPK and NF-κB p65↓	2015, Japan	(165)
*Lactobacillus Paracasei*	Mice	M2↑	TLR-4, NOX-4↓; MCP-1, PPAR-γ↓	2015, Korea	(153)
Astaxanthin	Patient samples, mice, cells	M1↓; M2↑	p38 MAPK↓, NF-κB↓, JNK↓	2015, Japan	(166)
*Opuntia ficus indica* seed	Mice	M2↑	PPAR-α↑; PPAR-γ↓; TLR4/NF-κB pathway↓	2016, South Korea	(163)
Glucoraphanin	Mice, cells	M1↓; M2↑	Nrf2 ↑; JNK↓; ERK↓; NF-κB p65↓	2017, Japan	(151)
Honokiol	Mice	M2↑	PPAR-γ signaling↑	2018, China	(162)
Retinoic-acid-related orphan receptor α	Mice, cells	M2↑	Kruppel-like factor 4↑	2017, Korea	(168)
Saxagliptin	Rats, cells	M1↓; M2↑	CaMKKβ/AMPK pathway↑; NF-κB↓	2018, China	(157)
Mucosal-associated invariant T cells	Patient samples, mice, cells	M2↑	MHCI-related molecule↑; IL-4↑	2018, China	(169)
IL-25	Mice, cells	M2a↑	IL-13/STAT6 pathway↑	2019, China	(170)
Raptor	Patient samples, mice, cells	M2↑	Dynamin-related protein 1↑	2019, China	(171)
*Ribes nigrum*	Mice	M1↓	IL-1β, TNF-α↓; miR-122-5p, miR-192-5p↓	2019, America	(172)
Liraglutide	Mice, cells	M2↑	cAMP-PKA-STAT3 signaling pathway↑	2019, China	(156)
Fermented Korean red ginseng	Mice, cells	M2↑	mTOR complex 1 signaling↓	2019, Korea	(173)
Rosiglitazone	Cells, mice	M1→M2	PPARγ↑; TLR4/NF-κB signaling pathway↓	2020, China	(155)
Myricetin	Mice, cells	M1↓; M2↑	TREM-1-TLR2/4-MyD88 signaling↓; NF-κB↓; p-STAT3↓	2020, China	(164)
Eccentric exercise and Caloric restriction	Mice	M1↓; M2↑	MCP1, TNF-α, IL-1β, IL-6↓; IL-10↑	2020, China	(174)
Anagliptin	Mice, cells	M1↓; M2↑	Dipeptidyl peptidase-4↓; NF-κB p65, p38 MAPK, ERK, JNK↓	2020, Japan	(175)
Annexin A5	Mice, cells	M1→M2	Pyruvate kinase M2↓	2020, China	(12)
Amlexanox	Cells	M2↑	TBK1/IKKϵ-NF-κB signaling pathway↓; IRF3 pathway↓	2020, Korea	(176)
Metformin and Genistein/Chlorogenic	Mice	M2↑	NF-κB↓; p-AMPK↑	2020, Iran	(159, 160)
Dasatinib	Mice	M2↑	COX2, SREBP-1, p-PDGFR↓; NF-κB↓	2021, Egypt	(167)
CD4 derived double negative T cells	Mice, cells	M1↓	TLR4, CCR2, TNF-α↓	2021, China	(177)
G protein-coupled bile acid receptor 1	Patient samples, mice, cells	M1↓	NLRP3 inflammasome activation↓	2020, China	(178)
**Aggravating MAFLD/NASH**					
Histone methyltransferase Suv39h2	Patient samples, mice, cells	M1↑	Sirt1↓; NF-κB↑; PPAR-γ↓	2017, China	(179)
CD44	Patient samples, mice	M1↑; M2↓	MCP-1/CCL2/CCR2↑	2017, France	(180)
MiR-141/200C	Patient samples, mice, cells	M1↑; M2↓	P-AMPK/AKT/GSK3↓; TLR4, p-mTOR/4EBP1↑	2017, America	(181)
P62-positive inclusion body	Patient samples	M1↑	Nrf2↑	2018, Japan	(148)
HSPA12A	Patient samples, mice, cells	M1↑	Nuclear M2 isoform of pyruvate kinase↑	2019, China	(182)
Iron overload	Mice, cells	M1↑; M2↓	KLF4↓, STAT6↓	2019, America	(183)
P38α	Patient samples, mice	M1↑	TLR4↑; TNF-α, CXCL10, IL-6↑	2019, China	(184)
Cholesterol	Patient samples, cells	M1↑	Exosomal miR-122-5p↑	2020, China	(185)
Hepatocyte-derived exosomal miR-192-5p	Patient samples, rats	M1↑	Rictor↓; p-Akt/p-FoxO1↓; FoxO1↑	2020, China	(186)
*E. coli* NF73-1	Patient samples, mice, cells	M1↑	TLR2/NLRP3 pathway↑; mTOR-S6K1-SREBP-1/PPAR-α signaling↑	2020, China	(154)

### 4.5 Liver Fibrosis

LF refers to the abnormal proliferation of intrahepatic connective tissue caused by various pathogenic agents, characterized by excessive deposition of extracellular matrix (ECM) (187). LF is essentially an excessive reparative response of the liver against chronic injury, and the formation mechanism is quite complex, which involves not only multiple cells, but also the complex cytokine network constituted by cellular autocrine and paracrine components (188). The activation of hepatic stellate cells (HSCs) is the major source of ECM as well as the central link of LF formation (189). The initiation and persistence of HSC activation is directly regulated by hepatic macrophages. On the one hand, macrophages activate HSCs and promote the progression of LF (190). On the other hand, during the reversal of LF, macrophages can drive HSC apoptosis and ECM degradation (191). Beljaars et al. (192) localized and quantified the macrophages with different phenotypes in the liver of humans and mice with LF. The results demonstrated that the numbers of both M1 and M2 macrophages were significantly increased during LF formation phase (192). However, compared with M2 macrophages, M1 macrophages may play a more important role in the regression of LF (192). In addition, Xi et al. (193) found that activated HSCs could promote hepatic macrophage infiltration through the CCL2/CCR2 pathway and induce M2 polarization to aggravate liver fibrosis. These studies amply suggest that macrophage polarization occupies an important role in LF progression.

Because there are various factors causing LF, and the pathogenesis and pathological process of different types of LF are different, the effects of macrophage polarization also vary. A study has shown that M1 macrophages and related pro-inflammatory cytokines are markedly increased in carbon tetrachloride (CCl_4_)-induced LF, whereas M2 macrophage polarization seems to predominate in schistosome infection-induced LF (194). Therefore, the inhibition of M1 and M2 macrophage polarization may respectively alleviate CCl_4_- and schistosome infection-induced LF. For example, margatoxin reduced M1 and increased M2 macrophage polarization by inhibiting STAT1 and promoting the phosphorylation of STAT6, which down-regulated pro-inflammatory cytokines and increased IL-10 expression, alleviating CCl_4_-induced LF in mice (195). Corilagin inhibited M2 polarization by inhibiting IL-13Rα1 signaling pathway, which alleviated schistosome egg-induced LF (196). However, this is not a completely uniform law. Ma et al. (14) showed that M1 polarization rather than M2 polarization contributed to the reduced activation and number of HSCs in the CCl_4_ and bile duct ligation-induced LF mouse model. Similarly, 2-methoxyestradiol alleviated CCl_4_-induced LF in mice by reducing macrophage infiltration and M2 polarization (197). These findings sufficiently indicate the complex association between LF and macrophage polarization.

The mechanisms by which macrophage polarization regulates LF are complex, involving multiple pathways and signaling molecules. Among them, Notch signaling pathway is closely related to multiple cellular activities, and has an important role in LF by regulating HSC activation and macrophage polarization (198). Bansal et al. (199) experimentally demonstrated that Notch pathway inhibition contributed to reducing M1 and promoting M2 macrophage polarization, as well as reducing the activation of HSCs and fibroblasts. Similarly, capsaicin and quercetin could also attenuate CCl_4_-induced hepatic inflammation and fibrosis in mice by inhibiting M1 polarization *via* the inhibition of the Notch signaling pathway (76, 200). However, in schistosome infection-induced LF, Notch inhibition also suppressed M2 macrophage polarization, thus ameliorating LF (77). Furthermore, IL-4Rα plays a dual role in LF. On the one hand, IL-4Rα activation induced M2 macrophage polarization to accelerate the process of LF (201). On the other hand, IL-4Rα-mediated STAT6 phosphorylation induced the production of MMP-12, contributing to hepatic fibrinolysis (201).

Currently, MSC transplantation is a promising anti-fibrotic strategy (202). Watanabe et al. (203) showed that MSCs could induce the M2 polarization of bone marrow-derived macrophages to play a synergistic role in the reversal of LF. Similarly, Luo et al. (204) showed that MSC transplantation could promote M2 and inhibit M1 macrophage polarization, as well as increase MMP13 expression and inhibit HSC activation, which exerted a synergistic anti-LF effect. Subsequently, further research has found that TNF-stimulated gene 6 is a major cytokine by which MSCs exert anti-fibrotic effects (205). Specifically, its liver-targeted delivery through calcium phosphate nanoparticles contributed to LF treatment, which was related to promoting M2 macrophage polarization and increasing MMP12 expression (205). Furthermore, in the development of LF, increased NKp46^+^ cells produced IFN-γ to induce M1 macrophage polarization, and limited M2 polarization and the production of pro-fibrotic factors, which inhibited the occurrence of metabolism-related LF (206). The role and mechanisms of macrophage polarization in LF are shown in **Table 6**.

**Table 6:** The role and mechanisms of macrophage polarization in LF.

Regulation Factor	Research Objects	Macrophage polarization	Mechanisms	Year and Country	Reference
**Alleviating LF**					
γ-secretase inhibitor	Patient samples, cells, CCl_4_-treated mice	M1↓	Notch signaling↓	2015, Netherlands	(199)
	Cells, schistosome-infected mice	M2↓		2016, China	(77)
NKp46^+^ cells	MCD-fed mice, cells	M1↑	IFN-γ↑	2016, America	(206)
*Toxoplasma gondii* GRA15_II_	Cells, schistosome-infected mice	M1↑	NF-κB↑; p-p38 MAPK↓; MMP-13↑; TGF-β1↓	2018, China	(207)
Lentiviral GRA15_II_				2018, China	(208)
Corilagin	Schistosome-infected mice	M2↓	SOCS1, KLF, PPARγ, PPARδ↓; IL-13/STAT6 signaling pathway↓	2016, China	(209)
Phosphatase and tensin homolog deleted on chromosome 10	CCl_4_-treated mice, cells	M2↑	PI3K/Akt/STA T6 signaling↑	2017, China	(210)
S-allyl-glutathione	CCl_4_-treated rats, cells	M1↓; M2↓	heat shock protein 47↓	2018, Japan	(211)
	DMN-treated rats, cells	M2↓			
Quercetin	CCl_4_-treated mice, cells	M1↓	Notch1 pathway↓	2018, China	(200)
Proline–serine–threonine–phosphatase-interacting protein2	CCl_4_-treated mice, cells	M1↓; M2↑	STAT1↓; STAT6↑	2018, China	(212)
Bone marrow MSC transplantation	CCl_4_-treated mice, cells	M1↓; M2↑	MMP13↑; IL-10↑, ΤGF-β1↓; caspase-3↑	2019, China	(204)
Splenectomy	ConA-treated mice	M2↑	NF-κB p65/p50↓	2019, China	(213)
Recombinant Sj16 protein	Schistosome-infected mice, cells	M2↑	Arg-1, IL-10↑; Th1 response↓	2019, China	(214)
Margatoxin	CCl_4_-treated mice, cells	M1↓; M2↑	P-STAT1↓; p-STAT6↑	2020, China	(195)
TNF-stimulated gene 6	CCl_4_-treated mice	M2↑	P-STAT1/3, p-p65, p-Akt↓; NF-κB↓	2020, China	(205)
Endoplasmic reticulum stress	CCl_4_-treated rats, cells	M1↑	TNF-α↑; TNF-R1/caspase 8↑	2020, China	(215)
Capsaicin	CCl_4_-treated rats, cells	M1↓	Notch signaling↓; TNF-α↓	2020, China	(76)
IL-22	CCl_4_-treated mice, cells	M1→M2	Erk1/2 and Akt pathways↓; STAT3 pathway↑	2021, China	(216)
**Aggravating LF**					
Cytochrome P450 2E1	Patient samples, DEN-treated mice	M2↓	CD163/CD68 ratio↓	2019, China	(217)
PC3-secreted microprotein	Patient samples, CCl4-treated mice	M1↑	CCR2↑	2020, China	(218)
LncRNA Lfar1	Cells, CCl_4_ and BDL-treated mice	M1↑	NLRP3 inflammasome↑; NF-ĸB pathway↑	2020, China	(219)
Activated HSCs	Patient samples, cells	M2↑	CCL2/CCR2 pathway↑	2021, China	(193)

### 4.6 Hepatocellular Carcinoma

Chronic liver diseases and the related inflammation may lead to ECM deposition to form LF, which if left unchecked, fibrous scars will continuously accumulate to form cirrhosis, ultimately causing HCC (220). HCC is the end stage of chronic liver diseases, and accounts for more than 80% of primary liver cancers worldwide, which is the fourth leading cause of cancer-related death (221). Although there are FDA-approved drugs for the clinical treatment of HCC currently, their therapeutic effects are quite limited. Therefore, it is still of great interest to explore the pathological mechanism of HCC and develop effective therapeutic drugs. Tumor growth, metastasis, and regression are influenced by the microenvironment in which it resides. Tumor-associated macrophages (TAMs) are major components of tumor microenvironment, and can differentiate into M1 and M2 phenotypes because of their plasticity, having an important role in the progression of HCC (222).

Autophagy is a key physiological and pathological process of the body, which is particularly important in tumor research (223). The study of Chang et al. (224) found that TLR2 ligands in HCC decreased NF-κB activity and promoted M2 macrophage polarization. Further research showed that HCC-derived high mobility group box 1 protein induced M2 polarization *via* the TLR2/NOX2/autophagy axis, promoting HCC development (225). These are perhaps potential targets for the treatment of HCC. Moreover, autophagy may influence the therapeutic effects of drugs on HCC. Tan et al. (226) found that baicalin promoted M2-TAMs to repolarize to M1 phenotype *via* autophagy-induced RelB/p52 activation, thus suppressing HCC. Sorafenib is currently an important option for the clinical treatment of HCC (227). However, Wei et al. (228) found that M2-TAMs could promote the autophagy of HCC cells and decrease the inhibitory effect of sorafenib on the proliferation of HCC cells, ultimately leading to sorafenib resistance in HCC treatment.

The latest evidence has suggest that lncRNAs play a crucial role in the occurrence and development of HCC (229). LncRNA TP73AS1 down-regulates miR539 to promote MMP8 expression, thus activating TGF-β1 signaling, which promotes M2 macrophage polarization and HCC progression (230). Similarly, lncRNA LINC00662 activates Wnt/β-catenin signaling to promote M2 polarization and HCC (231). In addition, angiogenesis is a prominent feature of tumor development (232). The study by Han et al. (233) showed that lncRNA-CRNDE could induce M2 polarization and promote angiogenesis, and the mechanism was associated with up-regulating the expression of JAK1, STAT6, AKT1, and angiogenesis-related proteins. Hou et al. (234) found that lncRNA MALAT1 expression was increased in HCC cells, which inhibited miR140 expression, ultimately promoting angiogenesis and M2 polarization, and enhancing immunosuppressive capacity.

Exosomes, the main members of EVs, have an important role in tumor initiation and development because of carrying and transmitting multiple biological signals (235). On the one hand, tumor-derived exosomes mostly accelerate tumor development. For example, lncRNA TUC339 of HCC-derived exosomes can promote M2 polarization, and its inhibition can promote the expression and phagocytic activity of M1 macrophage-related inflammatory factors (236). The transcription factor Sal like protein-4 can up-regulate miR-146a-5p expression of HCC-derived exosomes, promoting M2 macrophage polarization and the expression of the inhibitory receptor PD1 on the surface of T cells, which accelerates HCC progression (237). On the other hand, macrophage-derived exosomes can also regulate HCC progression. Wu et al. (238) showed that M2 macrophage-produced exosomes expressed specific CD11b/CD18 proteins, which up-regulated MMP-9 expression after entering HCC cells, thus promoting HCC migration. M1 macrophage-derived exosomal miR-326 inhibits NF-κB signaling pathway to inhibit the proliferation and migration of HCC cells (239).

The recurrence of HCC after tumor resection is a major hidden danger in HCC treatment. HCC prognosis contributes to the survival prediction of HCC patients, but there are limitations to traditional prognostic methods. Therefore, searching for HCC prognosis-related biomarkers is beneficial not only for better prognosis but also for the targeted therapy of HCC. Shu et al. (240) and Dong et al. (241) showed that M1/M2 macrophage polarization can be used as an independent prognostic factor for HCC, and the markers CD86 and CD206 can be used as the biomarkers for HCC prognosis. A subsequent study showed that GdCl3 could inhibit HCC progression in mice by down-regulating CD206 expression (242). In HBV-related HCC, T-UCR uc.306 expression is increased in M1 but down-regulated in M2 macrophages, which may serve as a HCC prognostic marker and a potential therapeutic target (243). Moreover, both neuromedin U and transmembrane205 can serve as the biomarkers for macrophage polarization-related HCC prognosis, which contributes to the targeted therapy of HCC (244, 245).

In conclusion, it is currently generally accepted that M2 macrophages induce tumor cell proliferation and metastasis to promote tumor development, whereas M1 macrophages have anti-tumor effects. In the HCC microenvironment, besides the role of HCC cells and macrophages, multiple internal factors and external interventions may also induce macrophage polarization to influence the development of HCC. For example, IL-37 inhibits M2 macrophage polarization mediated by the IL-6/STAT3 signaling pathway, thereby preventing HCC development (246). 4-methylumbelliferone can induce M1 macrophage polarization in the tumor microenvironment and inhibit the invasion of HCC cells (247). However, the study by Wang et al. (248) found that M1 macrophages could activate NF-κB/focal adhesion kinase signaling to contribute to HCC metastasis. In addition, the study by Zong et al. (249) demonstrated that M1 macrophages induced the high expression of the programmed cell death-ligand 1 in HCC cells by up-regulating IL-1β. Their findings support the pro-HCC effect of M1 macrophages. Therefore, the role of macrophage polarization in HCC is not as simple as it appears, and more in-depth studies are needed in the future. The role and mechanisms of macrophage polarization in HCC are shown in **Table 7**.

**Table 7:** The role and mechanisms of macrophage polarization in HCC.

Regulation Factor	Research Objects	Macrophage polarization	Mechanisms	Year and Country	Reference
**Alleviating HCC**					
Cantharidin	Cells, mice	M2→M1	STAT3↓; miR-214↑; β-catenin↓	2014, China	(250)
Baicalin	Cells, mice	M2→M1	RelB/p52 pathway↑	2015, China	(226)
GdCl_3_	Patient samples, mice, cells	M2↓	E-cadherin↑; N-cadherin, TWIST, Snail↓; CD206↓	2015, China	(242)
IL-12	Cells, mice	M1↑	STAT3/C-Myc pathway↓	2016, China	(251)
ToxoGRA15_II_	Cells, mice	M1↑	MMP-9, MMP-2↓; IL-6, IL-10↓; TNF-α, IL-12↑	2017, China	(252)
LncRNA cox-2	Cells, mice	M1↑; M2↓	p50/p65↑; COX-2↑	2018, China	(253)
MiR-98	Cells	M2→M1	TNF-α, IL-1β↑; TGF-β, IL-10↓; EMT↓	2018, China	(254)
SPON2	Patient samples, mice, cells	M1↑	Integrin-Rho GTPase-Hippo pathways↑	2018, China	(255)
Cryptotanshinone	Cells, mice	M1↑	TLR7/MyD88/NF-κB signaling pathway↑	2019, America	(256)
Sirtuin 1	Patient samples, cells	M1↑	NF-κB pathway↑	2019, China	(257)
Sirtuin 4	Patient samples, cells, mice	M2↓	FAO-PPARδ-STAT3 signaling pathway↓	2019, China	(258)
IL-37	Patient samples, cells, mice	M2→M1	IL-6/STAT3 pathway↓	2020, China	(246)
Retinoic acid-inducible gene I	Patient samples, cells, mice	M1↑	MAVS/TRAF2/NF-κB pathway↑	2020, China	(259)
4−methylumbelliferone	Mice, cells	M1↑	IL-6↓; TLR4, CD47, Sox2↓	2021, Australia	(247)
*G. lucidum* spore polysaccharide	Cells, mice	M1↑	TNF-α, IL-1β, IL-6, TGF-β1↑; PI3K/AKT pathway↑	2021, China	(260)
MiR-144/451a cluster	Patient samples, mice, cells	M1↑	hepatocyte growth factor (HGF)↓; migration inhibitory factor (MIF)↓	2021, China	(261)
**Aggravating HCC**					
Oxidored-nitro domain-containing protein 1	Patient samples, mice, cells	M2↑	Arg1, IL-10↑; IL-6, NF-κB↑	2018, China	(262)
N-myc downstream-regulated gene 2	Cells, mice	M1↓	NF-κB signaling pathway↓	2018, China	(263)
Wnt ligands	Patient samples, mice, cells	M2↑	Wnt/β-catenin signaling↑	2018, China	(15)
Neurotensin	Patient samples, cells, mice	M2↑	IL-8↑; MAPK and NF-κB pathways↑	2018, China	(264)
Sal-like protein-4	Patient samples, cells, mice	M2↑	Exosomal miR-146a-5p↑	2019, China	(237)
Intestinal dysbacteriosis	Patient samples, cells, mice	M2↑	IL-25↑; CXCL10↑	2019, China	(265)
LncRNA LINC00662	Patient samples, cells, mice	M2↑	Wnt/β-catenin signaling↑	2020, China	(231)
LncRNA MALAT1	Patient samples, cells, mice	M2↑	MiR-140↓; VEGF-A↑	2020, China	(234)
Extracellular ubiquitin	Patient samples, cells, mice	M2↑	CXCR4/ERK signaling pathway↑	2020, China	(266)
LncRNA TP73-AS1	Patient samples, cells, mice	M2↑	MiR-539↓; MMP8↑; TGF-β signaling↑	2020, China	(230)
Nogo-B	Patient samples, cells, mice	M2↑	Yes-associated protein (Yap)/transcriptional coactivator with PDZ-binding motif (Taz)↑	2020, China	(267)
High−mobility group box 1	Cells, mice	M2↑	TLR2/NOX2/autophagy axis↑	2020, China	(225)
Arsenite	Cells, mice	M2↑	MiR-15b↑; large tumor suppressor kinase 1↓; Hippo pathway↓	2021, China	(268)
lncRNA-CRNDE	Cells, mice	M2↑	JAK1/STAT6, AKT1↑; Notch1↑	2021, China	(233)
Cancer−associated fibroblast	Cells	M2↑	CXCL12↑; plasminogen activator inhibitor−1↑	2021, Japan	(269)
Cyclooxygenase-2	Patient samples, mice, cells	M2↑	TGF-β-Smad2/3↑; FoxP1↑	2021, China	(270)
Epithelial cell transforming 2	Patient samples, cells, mice	M2↑	PLK1/PTEN pathway↑	2021, China	(271)
Distal-less homeobox 6 antisense 1	Patient samples, cells, mice	M2↑	MicroRNA-15a-5p↓; CXCL17↑	2021, China	(272)

## 5 Discussion and Conclusion

**Figure 3:**
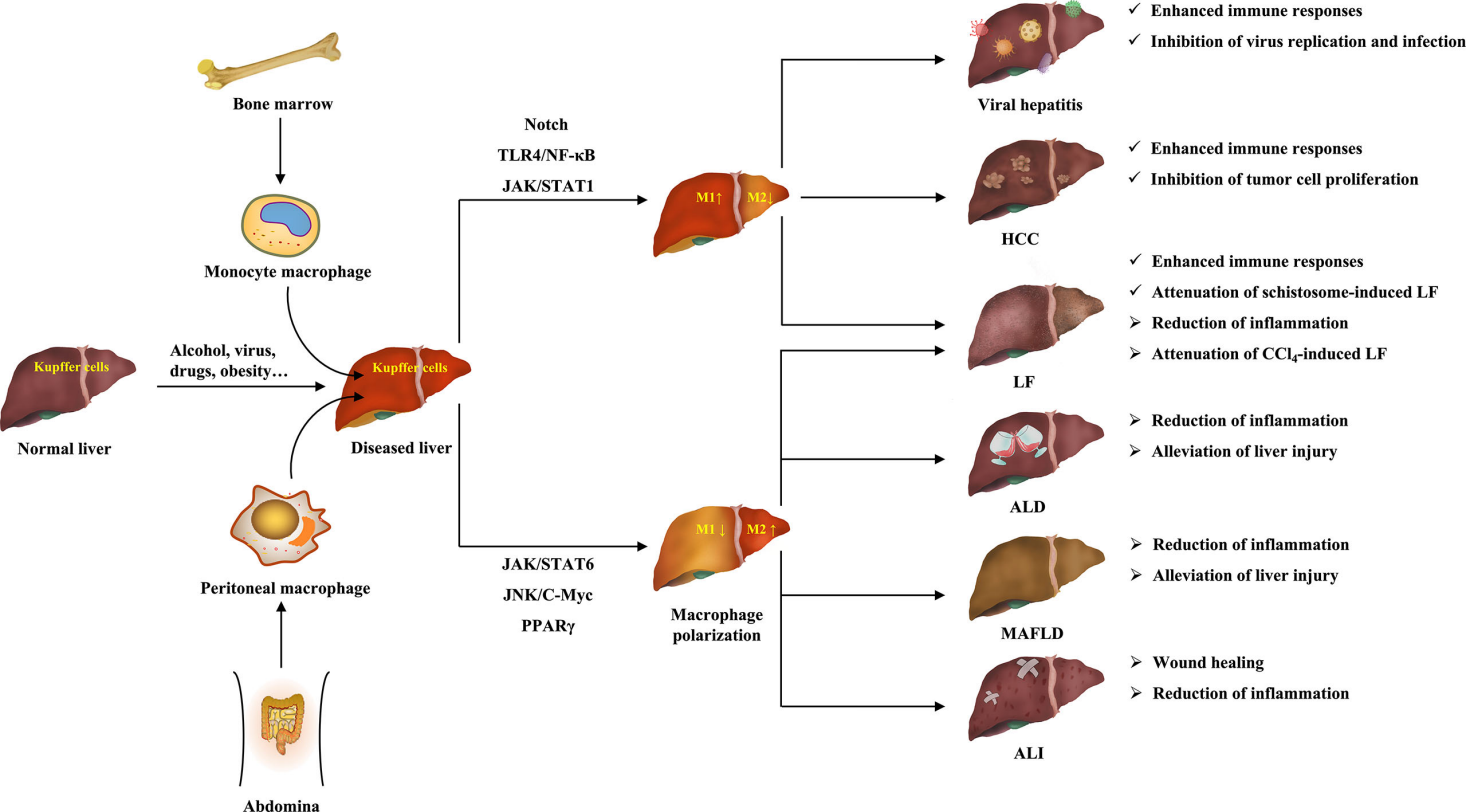
The ameliorative effects of macrophage polarization on various liver diseases.

Liver diseases are a major public health problem worldwide with high morbidity and mortality (5). At present, in-deep research on the pathogenesis of liver diseases and the development of effective drugs are important means for the treatment of liver diseases, as well as an important task and difficulty for scientific researchers. It is worth mentioning that recent studies have revealed that macrophage polarization plays an important role in the initiation and progression of liver diseases, and has a dual regulatory role on various liver diseases (273). This suggests that macrophages can influence the progression of multiple liver diseases through their polarization. **Figure 3** clearly shows the ameliorative effects of macrophage polarization on various liver diseases.

Hepatic macrophages have significant heterogeneity, which are composed of macrophages from multiple origins. Current studies have shown that macrophages mainly polarize to two phenotypes, pro-inflammatory M1 and anti-inflammatory M2 (25). Specifically, after being induced by their respective activators, M1 and M2 macrophages produce a large number of pro-inflammatory or anti-inflammatory cytokines and chemokines, thus acting on different targets to activate the signaling pathways associated with multiple pathological processes, which exert their regulatory functions (25). According to the data, macrophage polarization is mainly associated with TLR4/NF-κB, JAK/STATs, TGF- β/Smads, PPARγ, Notch, and miRNA signaling pathways. In addition, other signaling pathways, such as MAPK, mTOR, and so on, may be also involved. Therefore, targeting these signaling pathways may modulate macrophage polarization to alter the role of macrophages in liver diseases.

ALI refers to hepatic acute injury and hepatocellular necrosis mostly resulting from chemical drugs and poisons (86). Current studies generally show that M1 macrophage-secreted pro-inflammatory cytokines aggravate ALI, whereas M2 macrophages have the function of promoting tissue injury repair and secrete anti-inflammatory cytokines, which is beneficial for inflammation resolution and ALI alleviation. Therefore, promoting macrophage polarization to M2 phenotype and inhibiting M1 phenotype are important mechanisms to ameliorate ALI.

M1 and M2 macrophages in viral hepatitis have a complex cytokine profile (113). Specifically, after HBV and HCV infection, M2 macrophage polarization and IL-10 secretion are increased, and the pro-inflammatory factors produced by M1 macrophages are decreased, while some of them contribute to the alleviation of viral infection (274). Therefore, the inhibition of M2 macrophage polarization and the promotion of M1-associated cytokine production contribute to the alleviation of HBV- and HCV-related hepatitis. Differently, Labonte et al. (118) infected the mice with recombinant adenovirus expressing ovalbumin and performed an *in vitro* study of macrophage polarization, which showed that liver resident Kupffer cells polarized to M2 phenotype through up-regulating the expression of scavenger receptor AI, contributing to the reversal of chronic inflammation and tissue damage caused by viral infection. This suggests that the polarization of macrophages of different origins in the liver under the background of virus infection may have different effects on the disease development.

Ethanol leads to M1 macrophage polarization by acting on multiple receptors and targets in the body, thus aggravating inflammatory responses, which is one of the important pathogenesis of ALD (126). Current studies generally agree that M1 macrophages promote inflammation and aggravate ALD, whereas M2 macrophages have anti-inflammatory effects and attenuate alcohol-caused liver injury. Therefore, the development of targeted agents that induce macrophage polarization from M1 to M2 is of great significance for the clinical treatment of ALD. However, a study showed that TGF-β, a M2-associated profibrotic factor, was highly expressed in the liver of alcoholic hepatitis patients, which involved the interaction of M1 and multiple subtypes of M2 macrophages (M2a, M2b, and M2c) (275). Therefore, the role of macrophage polarization in ALD warrants further in-depth investigation.

MAFLD is a kind of liver injury closely related to metabolism and genetics with multiple complications, initially characterized by fat accumulation and degeneration, and probably develops into NASH with massive inflammatory responses (144, 145). Research showed that M1 macrophage polarization increased markedly during the development of MAFLD and NASH (146, 276). In addition to the body’s genes and receptors, many drugs can target relevant signaling pathways to inhibit M1 and promote M2 macrophage polarization, thus attenuating hepatic steatosis and inflammation in MAFLD/NASH. Interestingly, based on clinical studies and life experience, daily exercise and a controlled diet contribute to the improvement of MAFLD, which was shown to be related to the regulation of macrophage polarization (174). In addition, the study by Wan et al. (277) found that Kupffer cells with M2 phenotype could promote the apoptosis of M1 macrophages, inhibiting alcohol and high-fat diet-induced liver injury and inflammation. This is perhaps a potential mechanism by which M2 macrophages ameliorate ALD and MAFLD.

LF is a chronic liver disease resulting from the excessive repair of liver tissue injury, characterized by excessive deposition of ECM in the liver, which manifests as intrahepatic connective tissue dysplasia (278). Due to extensive etiologies, such as common CCl_4_ and schistosome infection in research, the pathogenesis of LF is not consistent (279). Therefore, different phenotypes of macrophages have different effects in different types of LF. In general, M1 macrophages promote CCl_4_-induced LF but inhibit schistosome infection-induced LF, but the effect of M2 macrophages is reversed. However, an increase in M1 polarization or a decrease in M2 polarization sometimes contributes to the alleviation of CCl_4_-induced LF (14, 197). Multiple mechanisms are involved in macrophage polarization in LF. Research proved that Notch signaling inhibition could alleviate CCl_4_- and schistosome-induced LF by inhibiting M1 and M2 polarization, respectively. More importantly, MSC transplantation, commonly used in LF treatment, also relies on the regulation of macrophage polarization. Furthermore, the study by Takemura et al. (211) found that s-allyl-glutathione reduced HSC activation in the rats with CCl_4_-induced LF by regulating macrophage polarization, rather than directly acting on HSCs. Therefore, macrophage polarization may have an indispensable role in LF.

HCC, the end stage of various chronic liver diseases, is a major cause of cancer-related death worldwide (221). Macrophages in the tumor microenvironment have a dominant role in cancer development and prognosis (280). The effect of macrophage polarization on HCC progression involves multiple cytokines and signaling pathways, including autophagy, lncRNAs, miRNAs, and the classical pathways of macrophage polarization. Exosomes serve as important vehicles for the signal transduction of HCC cells and macrophages. In general, M2 macrophages promote the proliferation and migration of HCC cells, whereas M1 macrophages can inhibit HCC development. However, some studies have shown that M1 macrophages may also have pro-tumor effects, indicating that the macrophage polarization in HCC is not restricted to pro-tumor M2 and anti-tumor M1 (248, 249).

In addition to the liver diseases discussed above, autoimmune liver disease is a group of special chronic liver diseases caused by immune dysfunction in the body, including autoimmune hepatitis (AIH), primary biliary cholangitis (PBC), primary sclerosing cholangitis (PSC) and their overlap syndrome (281). Interestingly, soluble CD163, a marker of macrophage activation, is closely associated with the severity of AIH, PBC and PSC, which can be used as a prognostic marker for them (282–284). Guicciardi et al. (285) further experimented to directly demonstrated that M1 and M2 macrophages were involved in the pathogenesis of PSC. These suggest us that it is worthy and necessary to pay more attention to the role of macrophage polarization in autoimmune liver disease. Li et al. (286) found that M1 macrophage polarization and the self-renewal of hepatic progenitor cells were increased in the livers of patients with PSC, associated with enhanced expression of Notch signaling pathway. Consistently, Jiang et al. (287) used 3,5-diethoxycarbonyl-1,4-dihydrocollidine to induce a liver disease similar to sclerosing cholangitis, and found that M1 polarization of macrophages led to decreased Wnt secretion and aggravated liver injury. A later study further found that cholangiocyte-derived exosomal lncRNA H19 exacerbated the inflammatory responses in patients with PBC and PSC by promoting Kupffer cell activation and M1 polarization of bone marrow-derived macrophage (288). Moreover, in a Con A-induced AIH mouse model, IL-34 and splenectomy were shown to inhibit inflammation and fibrosis, both of which were related to promoting M2 macrophage polarization (213, 289). From these findings, it is concluded that M1 macrophage polarization aggravates autoimmune liver disease and inflammation, while increased M2 polarization contributes to their remission. This can also be understood from the fact that M2 macrophages have immunoregulatory functions.

In addition, the pathogenesis of liver disease is complex, at least partly due to the crosstalk between the liver and peripheral tissues. Especially in fatty liver, the crosstalk of the liver with peripheral organs through the adipose tissue-liver axis, gut-liver axis, bone marrow-liver axis and brain-liver axis significantly influences the disease progression (290, 291). Altered gene expression in liver cells (hepatocytes and hepatic nonparenchymal cells) affects lipogenesis, lipolysis and inflammation in other tissues, and the metabolic and genetic changes in adipocytes and enterocytes also affect hepatic steatosis and inflammation (291). The growing of adipose tissue is accompanied by macrophage infiltration as well as the production of adipokines (leptin, adiponectin, resistin, etc.) and multiple cytokines, leading to inflammation, ECM accumulation and even insulin resistance, which in turn induces lipolysis and excessive free fatty acids release into the circulation, thus together contributing to hepatic steatosis after entering the liver (292). For example, adiponectin, an adipokine, promotes fatty acid oxidation in hepatocytes as well as reduces TNF-α and IL-10 production in Kupffer cells, contributing to the attenuation of hepatic steatosis and inflammation (293). Gut-liver crosstalk mainly results from increased intestinal permeability causing PAMPs to enter the liver *via* the portal circulation, also involving the changes of gut microbiota and the secretion of auxins, hormones, and bile acids (292). The interaction of bone marrow and the liver is mainly manifested by the recruitment of bone marrow-derived macrophages by activated Kupffer cells and HSCs in the liver, which have a regulatory role (291). In addition, the hypothalamic arcuate nucleus is the first-order neuron of the action of peripheral metabolic hormones (leptin, insulin, etc.), and the central nervous system controls energy balance to regulate obesity and fatty liver (290). Moreover, in other liver diseases such as ALI, viral hepatitis, and HCC, the presence of crosstalk between the liver and peripheral organs has been demonstrated (294–296). These suggest that the crosstalk between the liver and other organs plays a key role in the pathogenesis of liver disease. Furthermore, it is not difficult to see the critical role of macrophages. Therefore, we speculate that targeting the polarization of macrophages in multiple organs rather than just in the liver may contribute to treating liver disease by modulating the crosstalk between the liver and peripheral tissues.

Taken together, macrophage polarization plays an important role in a variety of liver diseases. In general, M1 macrophages have antigen presentation, pathogen clearance, and anti-tumor functions, having a protective effect in viral hepatitis, parasitic infection-induced LF, and HCC. In contrast, M2 macrophages have an anti-inflammatory effect and promote wound healing, which can effectively ameliorate the liver diseases mainly characterized by inflammatory injury, such as ALI, ALD, MAFLD, and CCl_4_-induced LF. However, in some cases (e.g., different targets or species), the effects of M1 and M2 macrophages in liver diseases may be opposite to the above. It is worth mentioning that current research is almost exclusively focusing on the M1 and M2 phenotypes of macrophages, so more macrophage phenotypes closely associated with disease progression are expected to be uncovered and studied. Therefore, due to the dual complexity of macrophage polarization and liver disease pathogenesis, more in-depth research on the role and mechanism of macrophage polarization in different liver diseases are worthwhile and necessary.

## Author Contributions

CW, CM, and YL designed the paper and recommended a structure for the review. CW, CM, YG, YZ, and HZ wrote the initial draft and prepared figures. LG, KF, and YL helped to revise the manuscript. All authors contributed to the article and approved the submitted version.

## Funding

The work was supported by National Natural Science Foundation of China (No: 81891012, 81630101, and U19A2010), Sichuan Province Science and Technology Program (No: 2021JDRC0041), and Xinglin Scholar Research Premotion Project of Chengdu University of Traditional Chinese Medicine (No: CXTD2018019).

## Conflict of Interest

The authors declare that the research was conducted in the absence of any commercial or financial relationships that could be construed as a potential conflict of interest.

## Publisher’s Note

All claims expressed in this article are solely those of the authors and do not necessarily represent those of their affiliated organizations, or those of the publisher, the editors and the reviewers. Any product that may be evaluated in this article, or claim that may be made by its manufacturer, is not guaranteed or endorsed by the publisher.

## Abbreviations

Akt, protein kinase B; ALD, alcoholic liver disease; ALI, acute liver injury; AMPK, AMP-activated protein kinase; APAP, acetaminophen; Arg1, arginase 1; AIH, autoimmune hepatitis; cAMP, cyclic adenosine monophosphate; CCL_4_, carbon tetrachloride; D-GalN, D-galactosamine; ECM, extracellular matrix; EVs, extracellular vesicles; HBV, hepatitis B virus; HCC, hepatocellular carcinoma; HCV, hepatitis C virus; HFD, high-fat diet; HSC, hepatic stellate cell; IFN-γ, interferon-γ; IL, interleukin; iNOS, inducible nitric oxide synthase; IRF, interferon regulatory factor; JAK, Janus kinase; LF, liver fibrosis; lncRNA, long noncoding RNA; LPS, lipopolysaccharide; MAPK, mitogen-activated protein kinase; miRNA, microRNA; MMP, matrix metalloproteinase; MSC, mesenchymal stem cell; mTOR, mechanistic target of rapamycin; MyD88, myeloid differentiation factor 88; MAFLD, metabolic-associated fatty liver disease; NASH, nonalcoholic steatohepatitis; NF-κB, nuclear factor-κB; NLRP3, NOD-like receptor protein 3; PI3K, phosphatidylinositol-3-kinase; PKA, protein kinase A; PPAR, peroxisome proliferators-activated receptor; PBC, primary biliary cholangitis; PSC, primary sclerosing cholangitis; SOCS, suppressor of cytokine signaling; STAT, signal transducer and activator of transcription; TAA, thioacetamide; TAMs, tumor associated macrophages; TGF-β, transforming growth factor-β; TLR, toll like receptor; TNF-α, tumor necrosis factor-α.
